# Chats about Medical Museums

**Published:** 1887-08-20

**Authors:** 


### Chats about Medical Museums.

By One of the Crowd.

(*Continued from p. 313, vol. ii.*)

### IX.—The Middlesex Hospital.

The Museum of the Middlesex Hospital is not one of the largest in London, and in two or three departments of interest it cannot compete with some others. It appears to be conspicuously wanting in models and dissections, and some of those which it does possess are not of the very best. But it is an exceedingly interesting collection nevertheless. It has a large display of pathological preparations, and is rather exceptionally provided with such objects as appeal to the interest of those who are hardly qualified to appreciate the scientific value of the museum.

Among the first things to arrest the attention is a small series of wax models, similar to one previously alluded to in a notice of Guy's—models of eggs in various stages of embryological development. These are exceedingly well done. On one of the walls is a bust of Sir Charles Bell, and at the far end of the museum is the skeleton of his favourite horse, while down just beneath him are the skeletons of the two of his dogs immortalised in Landseer's well-known "Dignity and Impudence"—in their bony nakedness neither of them presenting very much of the special characteristics of which in their day they were respectively the types. Near these skeletons is a glass bottle, appended to which is the photograph of an individual who, if he cannot be said exactly to have immortalised, must at least be allowed to have distinguished himself by a singular attempt to blow out his brains. Usually, no doubt, persons who proceed to painful extremities of this kind are impelled by sudden and violent emotion. This worthy went to work with singular deliberation. He was a gasfitter, or something of the kind, and not possessing a pistol, set to work to make one out of a piece of iron pipe, one end of which he plugged up, and then charged the barrel with powder and an awkwardly shaped piece of lead. He did not succeed in blowing his brains out, but his amateur artillery was so far effective that it battered a considerable hole in his forehead. Several fragments of bone had to be removed, and they are preserved here in the bottle, together with the primitive weapon and the missile, the photograph outside testifying to the neatness and skill with which, after the *débris* had been cleared out, the cavity was covered over and the mischief concealed.

In a previous article allusion was made to collections of calculi removed from the human system. There is such a collection at the Middlesex, and among others, several that by the skill and daring of one surgeon have been removed from the kidneys of living persons. This is one of the latest developments of surgical skill. Down in one corner of the room in a large bottle is an object which does not at all revolt the feelings, because it merely resembles a piece of charcoal or burnt wood in the shape of the lower portion of a man's leg. As a matter of fact it is a man's leg which dropped off. It became very badly gangrened, and should have been amputated, but the owner of it refused to submit to the operation. When, however, the progress of the disease had deprived him of the lower portion of the leg he submitted to the inevitable. An operation was performed above the knee, and the patient's life was saved. On the other side of the room is the plaster model of a splendidly developed arm belonging to a noted prize-fighter, and not far from this is another plaster cast of an arm removed from a mendicant impostor, who is said for many years to have excited the sympathy of the benevolent by showing a stump from which apparently a hand had been lost. In reality, the man had by persistent bandaging doubled up the hand under the wrist, and had so crushed and squeezed it as to make it appear that he had entirely lost it. Of course the member became utterly useless, and when after a while the miserable creature apparently came to repent of his folly, and to be anxious to make some sort of use of his maimed limb, he had to submit to the removal of the fore-arm, in order that some useful appliance might be fitted to the stump.

Perhaps the oddest thing in the whole collection is the windpipe of a man who was choked by a mutton chop. The oral tradition is that the chop was stolen, and the thief tried hastily to bolt it in order to conceal the fact. It is a pity that this does not appear to be the true story, for it is decidedly better than the one in the catalogue, from which it appears that the owner of the windpipe ordered a mutton chop in an eating-house. It was brought to him, and shortly afterwards the waiter found him dead in his seat. A coroner's inquest brought in a verdict of death from heart-disease, but a medical student happened to dissect a portion of the body for some purpose of his own, and found the real cause of death in the mutton chop wedged tightly in the windpipe. A novelist who should have invented such an accident as a means of disposing of the villain of his plot and rescuing his heroine from impending doom, would have been regarded as a great simpleton. There, however, are the two passages—one for food, and the other for air—preserved in spirit in a glass bottle, and here is the veritable mutton chop still wedged in the wrong one. Difficult though it undoubtedly is to conceive of such an accident, it is not perhaps more difficult than to imagine a medical student capable of perpetrating so gruesome a joke as the stuffing of a mutton chop down a dead man's windpipe in order to have something curious to discover. But it must be confessed that a thorough cross-examination of that young gentleman would have been interesting. Perhaps it was made; at all events, no doubt seems to be entertained as to the genuineness of this very remarkable occurrence.

In a capacious glass case is a considerable collection of skeletons and preserved specimens of the lower animals, and opposite, in one corner of the room, is a two-headed calf, or the hide of it stuffed, the doubleheaded skeleton of the thing being shown on a shelf overhead. Near this object is a large bottle in which is preserved the actual brain of the murderer Greenacre —a brain, by the way, which science has learned to recognise as of rather an inferior order, though fairly large. But any interest one may feel in such a relic of so commonplace a criminal as Greenacre is entirely eclipsed by another "exhibit"—to use a very objectionable word that has been necessitated by an age of exhibitions—in the shape of a particularly white skull, with what looks to be a small cap on the top of it. It is a relic of the Indian Mutiny—the skull of one of the chiefs who distinguished himself, even in that frightful saturnalia of cruelty, by the barbarities he practised on the English. The small circular cap on the top of his head is the wretch's scalp, which English officers took from him after shooting him.

